# The Impact of Environmental Regulations on Trade Flows: A Focus on Environmental Goods Listed in APEC and OECD

**DOI:** 10.3389/fpsyg.2021.773749

**Published:** 2021-11-26

**Authors:** Zhe Dai, Yunzhi Zhang, Rui Zhang

**Affiliations:** ^1^Law School, Jinan University, Guangzhou, China; ^2^Department of Economics, Jinan University, Guangzhou, China; ^3^Laboratoire d'économie d'Orléans (LEO), University of Orléans, Orléans, France

**Keywords:** environmental goods, Z-score, environmental regulations, International trade, ECOLEX platform, JEL classification: F18, F64, Q58

## Abstract

This paper aims to investigate the impact of environmental regulations on trade patterns, in terms of trade in environmental goods listed in Asia-Pacific Economic Cooperation (APEC) and Organization for Economic Co-operation and Development (OECD). Environmental goods are defined here as those that enable sustainable growth and reduce pollution from human activity. For this reason, issues on environmental goods can be put at the forefront to analyze the linkage between environmental regulations and trade flows in the perspective of international institutions. Within the framework of a gravity model, panel data on 112 exporter countries and 53 importer countries is used at a bilateral level over the period of 1989–2013 to gain an understanding of this trade-environmental regulation nexus. This paper proxies the environmental policies by employing the z-score, which includes three comprehensive environmental indicators. The findings indicate that the strict environmental policies impede the trade, and this effect is greater in terms of the environmental goods listed in APEC compared to the ones listed in OECD. Finally, although these stringent environmental regulations reduce the trade flows, they can help to foster the demand for environmentally-friendly products.

## 1. Introduction

The considerable volume of production and transportation generated by trade activities has given rise to a number of global environmental problems: primarily, climate change and pollution. In an attempt to cut emissions and make production greener, the World Trade Organization (WTO) Doha Ministerial Declaration brought the importance of environmental goods and services to the forefront of a global negotiation. The concept of environmental goods is aimed at bringing about sustainable growth and reducing the pollution caused by human activity. Besides engaging in a global cooperation, countries should also take a proactive response by implementing environmental regulations.

Several issues can be addressed concerning the trade-environment nexus (Murshed et al., 2021; Nathaniel et al., 2021). A particularly critical issue is the effect of environmental policies on trade patterns; the impact can be viewed from two perspectives, through the lens of competition. On the one hand, free trade allows firms with lax environmental standards to produce at a lower cost. In order to have competitive prices in the market, local firms adopt lenient environmental standards enabling low production costs. This has an effect of a “race to the bottom” (Frankel, 2009). On the other hand, according to (Frankel, 2009), the environment can also “gain from trade” by an international “ratcheting up” of environmental standards. Free trade allows the spread of the concept of environmental protection through the use (import) of environmentally-friendly goods and the entry of multinational firms. This results in a “race to the top” as countries follow these high environmental standards.

As the concept of environmental goods is designed to protect the environment while promoting trade and development, the impact of environmental regulations on trade in environmental goods can be a channel through which the trade-environment nexus is analyzed. Some related research questions can thus be put forward: Can trade patterns be influenced by introducing strict environmental regulations? What is the impact of trade on environmental goods? To answer these questions, we employ a gravity model to study how environmental regulations affect trade patterns, by comparing the environmental goods and similar goods^1^.

The impact of environmental regulations on trade patterns has been widely discussed (Xu, 2000; Jug and Mirza, 2005; Nunez-Rocha and Turcu, 2019), with previous literature focused on the impact of such regulations on the trade of “dirty” goods (Tobey, 1990; Van Beers and Van Den Bergh, 1997). Few studies to date have analyzed the impact of environmental regulations on clean industries, such as environmental goods in our case. Focusing on the relationship between environmental policy and trade patterns, our paper aims to investigate whether the environmental policy could promote trade in environmental goods.

The contributions of this study to the literature are three fold. First, it complements the studies on the trade-environment nexus by underlining two comparisons: the impact of environmental regulations on the trade patterns of both environmental goods and its similar goods; and South-North trade, and North-North trade, and so forth. For instance, Cantore and Cheng (2018) mention only the difference between developed and developing countries. Second, this study confirms that environmental regulations do not boost trade and they even impede the trade in environmental goods. This result is in line with some studies (Jug and Mirza, 2005; Nunez-Rocha and Turcu, 2019) and is contrary to the study of Wang et al. (2016), which focuses on country-specific characteristics. Last but not least, this study analyzes whether trade patterns are influenced by the changes in environmental regulations. To the best of our knowledge, this is the first attempt to create such a dummy to understand the impact of environmental regulations. The result highlights the fact that the stringency of environmental policies in the country of origin has a stronger impact on its exports.

The goal of environmental goods is to promote both trade and sustainable development. With a well setting, trade in environmental goods is expected to reduce the poverty by the means of expanding production and diversifying industries (Claro et al., 2007). The definition of environmental goods differs according to the institutions in question. The Asia-Pacific Economic Cooperation (APEC) forum announced a list of 54 environmental goods, and assigned products to four categories: (i) renewable energy, for example, gas water heaters (HS6: 8419.11); (ii) environmental monitoring, analysis and assessment equipment, such as gas or smoke analysis apparatus (HS6: 9027.10); (iii) environmental-production (principally relating to solid and hazardous waste, waste-water management and air pollution control), for instance filtering or purifying machinery and apparatus for gases (HS6: 8421.39); and (iv) environmentally preferable products, such as multi-layered bamboo flooring panels (Sugathan, 2013). The Organization for Economic Co-operation and Development (OECD) has a long list of 198 environmental goods. This list includes the categories from the APEC classification as well as a category for clean technology. In this case, four groups are specified by pollution management; remediation and cleanup; cleaner technologies; and products and resources management (Steenblik, 2005).

The remainder of this paper is organized as follows: The second section presents the literature related to environmental regulations and their impact on trade patterns. The empirical methodology and the data are described in the third section. The fourth section reports the results of the estimations, while robustness checks are presented in the fifth section. The last section concludes.

## 2. Literature Review

With the existing studies, the literature review is considered to be split into two parts. First, we show the related theories at the theoretical level. Second, empirical studies are discussed.

### 2.1. Theoretical Framework

A general equilibrium model is built by Pethig (1976) for a two-sector, two-country assumption. This research reveals that environmental regulations can have an impact on comparative advantage in each country. Based on the Heckscher-Ohlin model, McGuire (1982) incorporates an environmental factor regarding environmental regulations, finding that environmental regulations generate a shift in production from more regulated countries to less regulated countries.

Later on, an interesting model is developed by Copeland and Taylor (1994) to examine the link between trade and the environment. They adopt an N-goods general equilibrium model in the case of North-South trade. In this setting, Northern countries with strict environmental policies are endowed with capital, which is considered to be the main factor in producing pollution-intensive industries. Moreover, the Southern countries with loose environmental regulations are endowed with labor, which is seen as the factor in the less-polluting industries. Therefore, under the condition of free trade, different outcomes may emerge. When the factor-endowment hypothesis applies, the North specializes in and exports “dirty” goods, while the South specializes in and exports “clean” goods. However, under the pollution haven hypothesis, environmental regulation is incorporated into trade patterns. The North specializes in and exports clean goods since the stringency of environmental regulations results in high production costs for dirty goods. This results in the transfer of dirty industries to the South.

One of the crucial branches of theoretical work studies the relationship between trade and the environment. In the long run, trade is considered to be the engine of growth and it has been seen as an influential factor in environmental quality. The seminal work of Grossman and Krueger (1991) states that trade can impact the environment through scale, composition, and technique effects. Furthermore, Copeland and Taylor (2005) complete their studies and develop an integrated theoretical framework. The three channels are explained as follows: First, holding constant the technology level and composition of industries, free trade introduces a high volume of economic activities, which will induce a high intensity of pollution. Second, at the same level of economic scale and with the same composition of industry, free trade attracts more multinational firms with advanced technology and sustainable management. Finally, countries that have pollution-intensive industries will produce and export “dirty” goods and have a greater pollution level at the same level of economic scale and technology.

### 2.2. Empirical Analysis

Since environmental problems and sustainable development has attracted considerable attention among the public, academic research discusses various related topics. Economic growth, human capital, energy consumption, environmental policies, etc. are expected to be the factors to affect the environment (Murshed et al., 2021; Nathaniel et al., 2021). Among these factors, trade is also an influential element on environment.

However, the impact of the trade on the environment remains inconclusive. Some studies find that trade has a positive impact on environmental quality (Antweiler et al., 2001; Frankel and Rose, 2005; among others). More recent research found that trade has an ambiguous impact on pollution level (Cole and Elliott, 2003; Managi et al., 2009; among others). Moreover, at the country-specific level, the trade-environment nexus is tackled for the case of China (Cole et al., 2011; Poncet et al., 2015; Zhang, 2020). Recently, a strand of literature considers a more comprehensive indicator to evaluate environmental quality, which is the “ecological footprints.” Nathaniel et al. (2021) use a sample of “Next Eleven” countries from 1990 to 2016 to analyze the impact of environmental regulations on ecological footprints. In their study, the control variable of international trade confirms that trade deteriorates environmental quality.

One sustainable goal can be set for understanding trade in environmental goods. Many related studies have also attracted large attention in recent years (Tamini and Sorgho, 2018; Zugravu-Soilita, 2019; De Melo and Solleder, 2020; Can et al., 2021). Zugravu-Soilita (2019) examines whether trade in environmental goods has an impact on environmental performance. Employing Instrument Variable (IV) and Generalized Method of Moments (GMM) methodology to control for endogeneity, this study analyzes 114 countries over the period from 1996 to 2011, reporting that trade in environmental goods has no impact on emissions in general. Therefore, this specific type of trade reduces pollution for the net exporters and has a detrimental effect on net importers. A different result is shown by Can et al. (2021). Based on the panel data from 2003 to 2016 that covers 35 OECD countries, the authors investigate the impact of a green openness index on environmental quality. In their paper, trade in environmental goods and environmentally preferable goods are considered to be a “green openness index.” They find that an increase in green openness can improve environmental quality.

Further on, Tamini and Sorgho (2018) pay attention to the trade cost of environmental goods. By investigating a sample of 167 countries that export to OECD countries, they find that the common gravity variables (such as common borders, common language, and a common legal system) promote trade in environmental goods. However, the trade cost elasticity of environmental goods is high in general, except for these high-income countries. They suggest analyzing the tariff and non-tariff measure's impact on environmental goods. To follow this insightful suggestion by Tamini and Sorgho (2018), further research by De Melo and Solleder (2020) disentangles these findings. De Melo and Solleder (2020) study the impact of tariff and non-tariff measures on environmental goods by employing the Poisson Pseudo Maximum Likelihood (PPML) methodology. They use a comprehensive index to proxy non-tariff measures: regulatory distance. Finally, these findings show that tariffs reduce trade in environmental goods; therefore, the regulatory distance increases trade in environmental goods.

Among the studies on sustainable goal, the impact of environmental regulations shows it highly importance:

First, some researches has found that environmental policy can affect trade patterns (Xu, 2000; Jug and Mirza, 2005; among others). Using a standard gravity model, Xu (2000) employs both time-series and cross-section methodologies to identify whether the stringency of environmental regulations has an impact on trade patterns. Based on data over the period from 1965 to 1995 for 34 countries, the results show that the stringency of environmental regulations has no impact on trade patterns. However, the dataset used in this paper is limited, and thus might not be convincing enough to explain this relationship. The impact of environmental policies on trade differs for individual countries. Jug and Mirza (2005) use a sample including 12 importing countries and 19 exporting countries in both Eastern and Western Europe from 1996 to 1999 to analyze the impact of environmental abatement costs on trade flows. To avoid the bias resulting from the correlation between residuals and abatement costs, they also estimate the model using a GMM methodology. Their results confirm a negative relationship between environmental abatement costs and trade. They also conclude that trade by countries in Eastern Europe is more sensitive to stringent environmental regulations than that of Western economies.

Second, a strand of research focuses on environmental regulations that have an impact on some specific trade related to the environment, namely “dirty” goods and energy goods (Tobey, 1990; Van Beers and Van Den Bergh, 1997; Nunez-Rocha and Turcu, 2019; among others). Tobey (1990) investigates the impact of rigorous environmental regulations on the trade of “dirty” commodities, which are defined as having high dependence on environmental resources. Using a sample of 23 countries, including 13 industrial nations and 10 developing countries from 1974 to 1980, the author finds no evidence to support the “Pollution Haven Hypothesis.” Therefore, the introduction of environmental controls does not impact the location of dirty industries. Similarly, using the same sample and the same environmental proxy variables, Van Beers and Van Den Bergh (1997) confirm the results of Tobey (1990). Applying further tests, they find that stringent environmental policy does have a significant negative impact on non-resource-based exports. Focusing on the specific case of trade in energy resources, Nunez-Rocha and Turcu (2019) use a sample of 141 countries from 1998 to 2015 to analyze how environmental laws impact trade in fuels. By dividing environmental laws into those regarding the extraction of natural resources and laws regarding their use, their paper shows that an increase in the number of laws or treaties related to energy, especially the ones related to energy use, reduces trade in fuel.

Third, an attempt to understand the relationship between environmental regulations and trade in environmental goods, by employing the gravity model, is conducted by (Cantore and Cheng, 2018). They investigate the impact of environmental taxes on trade in environmental goods. They find that the impact of environmental taxes is heterogeneous, and varies across developed and developing countries. The special case of China is provided by Wang et al. (2016). They apply Feasible Generalized Least Squares (FGLS) to study the impact of environmental policy on different categories of products. The findings of this study show that the impact of environmental regulations varies across product categories: strict environmental regulations decrease the exports of polluted goods but increase the trade of green products.

In sum, previous studies have widely discussed related topics on environmental regulations and trade in environmental goods. However, they do not discuss comprehensively the impact of environmental regulations on trade in environmental goods. Moreover, to the best of our knowledge, the existing studies do not analyze the impact of changes in environmental regulations on trade. This study is meant to fill this shortage.

## 3. Model Specification and Data

### 3.1. Environmental Policy Setting

To analyze the impact of environmental regulations on trade patterns, we need to find a suitable proxy for these regulations. There are a number of ways to quantify and proxy environmental regulations (Brunel and Levinson, 2016). For instance, they can be assessed in terms of government behavior, firm behavior, household behavior, and these can be based on environmental performances. Some researchers use a dummy variable for agreements on the environment, such as the Helsinki Declaration or the Oslo Accords (Managi et al., 2009). One strand of the literature uses indicators, such as energy intensity, abatement cost intensity, and survey indices for regulations. However, previous studies apply only one indicator to proxy environmental policy, which is not enough to describe the characteristics of environmental policy (Wang et al., 2016; Nunez-Rocha and Turcu, 2019; among others). Here, we use the z-score method proposed by Kheder and Zugravu (2012). Unlike proxies for environmental regulations that are based on a single variable, the z-score method allows us to comprehensively consider several factors together.

When we build the z-score index, three factors are included in order to evaluate environmental regulations for each country. The first factor is the cumulative number of all the pieces of environmental legislation ratified by a country. In their assessment of environmental regulations, Núnez-Rocha and Martınez-Zarzoso (2018) consider that the intensity of environmental laws in a country reflects that country's concern about pollution. To be precise, if a country passes multiple laws on environmental issues, this represents strong management and a desire by the government to protect the environment. However, environmental laws will have no impact on the environment if there is a low level of institutional enforcement (Bazillier et al., 2013). Hence, law and order should also be taken into account as a second factor to proxy the legal system (Núnez-Rocha and Martınez-Zarzoso, 2018). Moreover, a third factor, energy efficiency (GDP/total units of energy used), is used to capture regulations that are strongly related to energy (Kheder and Zugravu, 2012).

### 3.2. Methodology

In order to examine the impact of environmental policies on trade patterns, in terms of environmental goods and similar goods, we estimate a general gravity model used for analyzing bilateral trade relations by following the study of Anderson and Van Wincoop (2003). Since trade in environmental goods does not occur for every pair of the trading partners, we apply the Poisson Pseudo Maximum Likelihood (PPML) methodology (Silva and Tenreyro, 2006) to account for the zero trade flows. We further include remoteness variables to control for the issue of multilateral resistance terms (Head and Mayer, 2014). Remoteness is computed as the ratio of the bilateral distance between the origin and the destination, weighted by world GDP share. As argued in the paper by Mart́ınez-Zarzoso (2018), environmental provisions in trade agreements are also important for trade. We include a variable capturing environmental provisions for our estimation of trade in environmental goods. Our estimations are as follows:


(1) $$\begin{array}{l} Exports\_Similar_{ijt}=exp[\beta_{1}zscoreER_{it}+\beta_{2}zscoreER_{jt}\\ \;+\beta_{3}ln(GDP_{it})\\ +\beta_{4}ln(GDP_{jt})+\beta_{5}ln(Y_{it})+\beta_{6}ln(Y_{jt})\\ +\beta_{7}RTA_{ijt}+\beta_{8}ln(DIS_{ij})+\beta_{9}CON_{ij}+\beta_{10}ComCurrency_{ijt}\\ +\beta_{11}ComReli_{ijt}+\beta_{12}Normal\_Tariff_{ijt}+\beta_{13}Remoteness_{it}\\ \;+\beta_{14}Remoteness_{jt}+\gamma_{ij}+\sigma_{t}+]\times \varepsilon_{ijt} \end{array}$$

(2) $$\begin{array}{l} Exports\_EG_{ijt}=exp[\alpha_{1}zscoreER_{it}+\alpha_{2}zscoreER_{jt}+\alpha_{3}ln(GDP_{it})\\ \;+\alpha_{4}ln(GDP_{jt})+\alpha_{5}ln(Y_{it})+\alpha_{6}ln(Y_{jt})+\alpha_{7}RTA_{ijt}\\ +\alpha_{8}ln(DIS_{ij})+\alpha_{9}CON_{ij}+\alpha_{10}ComCurrency_{ijt}+\alpha_{11}ComReli_{ijt}\\ \;+\alpha_{12}EG\_Tariff_{ijt}+\alpha_{13}EnvirPro_{ijt}+\alpha_{14}Remoteness_{it}\\ \;+\alpha_{15}Remoteness_{jt}+\gamma_{ij}^{\prime }+\sigma_{t}^{\prime }]\times \varepsilon_{ijt}^{\prime } \end{array}$$

where *ln*(.) denotes the natural logarithm;

*Exports*_*Similar*_*ijt*_ is exports of similar goods, which are the products that share the same HS4 commodity code with environmental goods. Hereafter, we refer to this type of goods as similar goods (in terms of their similarity to environmental goods).

*Exports*_*EG*_*ijt*_ is exports of environmental goods. Trade flows are from origin *i* to destination *j* in year *t* in current US dollars;

*zscoreER*_*it*_, *zscoreER*_*jt*_ are the proxy for environmental policy based on several variables in year *t* for origin *i* and destination *j*, respectively;

*GDP*_*it*_, *GDP*_*jt*_ denotes GDP level from origin *i* to destination *j*;

*Y*_*it*_, *Y*_*jt*_ are country *i* and trading partner *j*'s GDP per capita in US dollars in year *t*;

*RTA*_*ijt*_ is a dummy variable for regional trade agreements that takes a value of 1 if the origin *i* has a regional trade agreement relationship with destination *j* and 0 otherwise. *DIS*_*ij*_ represents the geographical distance between origin *i* and destination *j*;

*CON*_*ij*_ is a dummy variable that takes a value of 1 if the origin *i* and destination *j* are contiguous, and 0 otherwise;

*ComCurrency*_*ijt*_ is a dummy variable for a common currency that takes a value of 1 if the origin *i* and destination *j* share the same currency, and 0 otherwise;

*ComReli*_*ijt*_ is a dummy variable that takes a value of 1 if the origin country *i* and destination country *j* have the same religious background, and 0 otherwise;

*Normal*_*Tariff*_*ijt*_ is the average tariff for similar goods between the origin *i* and destination *j*;

*EG*_*Tariff*_*ijt*_ is the average tariff for environmental goods between the origin *i* and destination *j*;

*EnvirPro*_*ijt*_ reports the number of clauses relating to environmental provisions in the trade agreement between the origin *i* and destination *j*.

*Remoteness*_*it*_, *Remoteness*_*jt*_ are the indicators used to control for multilateral resistance terms.

γ_*ij*_ denotes the fixed effects for each country pair, origin *i* and destination *j*; σ_*t*_ is a time dummy; ε_*ijt*_is the error term.

### 3.3. Data

The dataset used for the model above covers 112 exporting countries and 53 importing countries of environmental goods from both the APEC and OECD lists of environmental goods from 1989 to 2013, at the macro level. The z-score that is used to proxy environmental regulations (*ER*_*zscore*) consists of three indicators: the number of environmental laws, law and order, and renewable energy consumption.

For the first component, the number of environmental laws in the origin and destination (*LawNum*) is sourced from the ECOLEX platform jointly operated by the Food and Agriculture Organization (FAO), the International Union for Conservation of Nature (IUCN), and the United Nations Environment Program (UNEP). This database includes the laws, treaties, regulations, etc., in several areas related to the environment and sustainable development. Nunez-Rocha and Turcu (2019) analyze the impact of environmental laws on trade in fuel products by identifying laws on different subjects. Here, we simply aggregate all environmental laws to give the total number.

For the second component, we use a variable from the International Country Risk Guide (ICRG) rating dataset, namely, we use the law and order score to proxy the legal system (*LawOrder*) in each country. This score is based on both the strength and impartiality of the legal system and the awareness of the citizens. For the third component, renewable energy consumption (as a percentage of total final energy consumption) is taken from the World Bank dataset.

Environmental goods are those from the APEC list of 54 goods (Sugathan, 2013) and the OECD list of 198 goods (Steenblik, 2005); and similar goods are defined as those goods that share the same HS4 categories as the environmental goods. Data for the trade value of the environmental goods (*EG*) and similar goods (*Similar*) in US dollars are drawn from the BACI database. In addition, GDP (*GDP* in the regression), GDP per capita (*Y* in the regression), and the presence of a regional trade agreement (*RTA* in the regression) are gravity variables drawn from CEPII. Data on tariffs are from the World Integrated Trade Solution (WITS), and they are computed as the average for both environmental goods *EG*_*tariff* and similar goods (*Normal*_*tariff*). In addition, data on environmental provision (*EnvirPro*) are taken from the global preferential trade agreements database (World Bank). The data description is provided in Table 1.

**Table 1:** Data description.

**Variable**	**Observation**	**Mean**	**Std. dev**.	**Min**	**Max**
EG (APEC)	576,017	9911.235	163304.8	0	2.56E+07
EG (OECD)	576,017	14792.91	194620.1	0	1.86E+07
Similar (APEC)	576,017	9547.5	128514.5	0	1.56E+07
Similar (OECD)	576,017	20782.55	320525.1	0	3.05E+07
ER_zscore_it	300,226	−2.42E-18	1	−2.641038	4.836046
ER_zscore_jt	289,471	1.01E-17	1	−2.576211	4.866756
Lawnumber_it	482,335	45.26942	81.61643	0	1107
Lawnumber_jt	483,764	43.53712	80.19962	0	1107
LawandOrder_it	392,493	4.048302	1.372856	0	6
LawandOrder_jt	379,951	4.007559	1.378445	0	6
Energy_it	432,390	7.417811	3.681839	0.5473055	34.51366
Energy_jt	420,590	7.397313	3.715809	0.5473055	34.51366
lnGDP_it	513,619	24.52549	2.325269	15.99307	30.52343
lnGDP_jt	513,444	24.36071	2.407928	15.99307	30.52343
lnY_it	513,619	8.424272	1.611477	4.171462	11.52108
lnY_jt	513,444	8.373619	1.622193	4.171462	11.52108
RTA_ijt	544,921	0.123484	0.3289922	0	1
lnDIS_ij	544,921	8.670823	0.8130766	4.107106	9.897904
CON_ij	544,921	0.0206525	0.1422183	0	1
ComCurrency_ijt	544,921	0.0157417	0.1244747	0	1
ComReli_ijt	517,167	0.1774818	0.254848	0	1
Normal_tariff_ijt	392,792	7.879011	7.009051	0	105.36
EG_tariff_ijt	123,800	4.4133	4.863762	0	38.97279
EnvirPro_ijt	576,017	0.2406335	4.233867	0	131
Remoteness_it	576,017	0.6962814	3.666752	0	161.6643
Remoteness_jt	576,017	0.9706235	4.463354	0	173.0122

## 4. Empirical Results

This section presents the results for the impact of environmental policies on trade patterns, comparing trade in environmental and similar goods. To that end, we employ a PPML estimation based on the gravity model. First, the focus is on the relationship between environmental policy and the trade values of environmental goods and similar goods. Furthermore, the environmental policy-trade nexus is studied by examining the trade flows among different groups of countries.

### 4.1. Baseline

First, our dependent variable is the trade values of two types of goods in two lists, namely environmental goods on the APEC and OECD lists, and similar goods. The results indicate that environmental regulations have a negative impact on trade, as shown in Table 2. Although we consider a more comprehensive indicator to evaluate environmental regulations, this negative linkage is in line with the previous literature on exports of all products (Jug and Mirza, 2005) and fuel exports (Nunez-Rocha and Turcu, 2019). Column (1) reports the results of exports for similar goods (in terms of environmental goods on the APEC list). Although the variables for environmental regulation in both the origin and the destination countries indicate that stringent environmental policies impede trade, the magnitude of the coefficients indicates that policies have only a slight effect. The results for the environmental goods on the APEC list are shown in column (2). They show a negative, statistically significant effect for both origins; however, the magnitude is greater for the origins than for the destination. This indicates that strict environmental regulations in the origin countries are a greater hindrance to exports of environmental goods.

**Table 2:** Main results.

	**(1)**	**(2)**	**(3)**	**(4)**
	**Similar (APEC)**	**EG (APEC)**	**Similar (OECD)**	**EG (OECD)**
*lnGDP*_*it*_	0.0793	1.368***	−0.180+	0.508*
	(0.135)	(0.358)	(0.0991)	(0.239)
*lnGDP*_*jt*_	0.194	0.00311	−0.877***	0.0931
	(0.155)	(0.178)	(0.108)	(0.142)
*lnY*_*it*_	0.623***	−0.371	0.981***	0.331
	(0.138)	(0.373)	(0.103)	(0.247)
*lnY*_*jt*_	0.444**	0.732***	1.655***	0.516***
	(0.159)	(0.194)	(0.117)	(0.147)
RTA	−0.0225	−0.153**	0.0467	−0.164***
	(0.0285)	(0.0503)	(0.0298)	(0.0352)
*ComCurrency*_*ijt*_	−0.0197		−0.0779**	
	(0.0279)		(0.0263)	
*Normal*_*tariff*_*ijt*_	−0.0119***		−0.0258***	
	(0.00293)		(0.00449)	
*Remoteness*_*it*_	0.524*	2.981***	0.195+	0.847**
	(0.205)	(0.452)	(0.111)	(0.271)
*Remoteness*_*jt*_	−0.200	2.439***	0.409***	1.644***
	(0.123)	(0.259)	(0.104)	(0.131)
ERzscore_it	−0.0338*	-0.126***	-0.195***	−0.126***
	(0.0150)	(0.0273)	(0.0132)	(0.0201)
ERzscore_jt	−0.0241*	−0.0645**	−0.101***	−0.0624***
	(0.0113)	(0.0246)	(0.0125)	(0.0151)
*EG*_*tariff*_*ijt*_		−0.0178*		−0.0129***
		(0.00747)		(0.00344)
*EnvirPro*_*ijt*_		−0.00491***		−0.00178
		(0.00130)		(0.00111)
Constant	−4.998	−27.92***	17.45***	−11.51*
	(3.545)	(7.328)	(2.547)	(5.039)
Observations	104418	38986	115211	41531
IJ FE	YES	YES	YES	YES
year FE	YES	YES	YES	YES

Standard errors in parentheses. ***, **, *, + denote significance at 0.1, 1, 5, and 10% levels, respectively. Dependent variable: Similar denotes the export value of similar goods in terms of environmental goods; EG denotes the export value of environmental goods. Columns (1) and (2) are the goods in the list of the Asia-Pacific Economic Cooperation (APEC); columns (3) and (4) are the goods in the list for the Organization for Economic Co-operation and Development (OECD).

Furthermore, negative coefficients for regulations are presented in column (3) for the case of environmental goods on the OECD list. The elasticity of coefficients shows a higher impact than the ones on the APEC list. The results for environmental goods that are listed by the OECD are basically the same as the ones by the APEC in column (2). This finding reinforces the fact that stringent environmental regulations do not help a country trade more in environmental goods. This can be explained by the fact that strict policies on the environment will trigger local demand for environmentally-friendly commodities; thus, countries reduce their exports in order to supply the local market. However, this issue lies beyond the scope of this paper, since we do not have data on production activity.

In regard to the gravity variables, they show the coefficients have the expected signs and are generally statistically significant. Although GDP does not always show a positive impact on these types of products, in most cases GDP per capita shows a positive sign for both origin and destination countries. This finding indicates a higher demand for environmental goods and similar goods in countries with high income levels. The results for tariff report negative signs both for the case of similar goods and for environmental goods, indicating that tariffs impede trade in all cases. Furthermore, dummies such as for regional trade agreements and a common currency, have negative effects on trade. Surprisingly, environmental provisions show a negative impact on trade in environmental goods on the APEC list. Remoteness, which is the proxy used to control for the multilateral resistance terms, reports a positive sign and is statistically significant in all the estimations.

### 4.2. Trade Flows

Disparities in development levels compound the gaps in environmental policy between high-income (North) and low-income (South) countries. The latter group of countries is assumed to have laxer regulations, in terms of environmental protection. On the contrary, it is understood that high-income countries implement strict environmental policies. In a previous study, Cantore and Cheng (2018) discuss the impact of environmental taxes on trade in environmental goods by considering the difference between developed and developing countries. Therefore, no investigation underlines the trade flows between different countries at income level. We assume that the North (with strict environmental policies) will export or import more environmental goods. In this subsection, we analyze the relationship between environmental policy and trade patterns by distinguishing four pairs of trade flows, namely North-South, South-North, North-North, and South-South trade flows. We identify the countries in the group of high-income level as North, while the rest are deemed as belonging to the South.

The summary for different trade flows is shown in Table 3^2^. In short, it can be seen that the stringency of environmental regulations has varied effects across the different trade flows. In the first pair, for the trade from North to South, the results are only statistically significant and positive for similar goods (relative to both the APEC and OECD lists). They indicate that strict environmental policies in the destination (the South, in this case) promote more trade in similar goods, but not trade in environmental goods. This result runs counter to our assumption. The next pair is the North-North trade. The results in the table show a negative sign for the coefficients of environmental regulations. These negative signs indicate that the environmental regulations hinder trade between rich countries.

**Table 3:** Trade flows.

	**(1)**	**(2)**	**(3)**	**(4)**
	**Similar (APEC)**	**EG (APEC)**	**Similar (OECD)**	**EG (OECD)**
**1.North-South**				
ERzscore_it	−0.0455	−0.0371	−0.242***	−0.0701*
	(0.0279)	(0.0630)	(0.0384)	(0.0345)
ERzscore_jt	0.0597**	−0.00232	0.0600**	0.000164
	(0.0190)	(0.0346)	(0.0225)	(0.0168)
**2.North−North**				
ERzscore_it	0.0248	−0.157**	−0.119***	−0.0922*
	(0.0223)	(0.0482)	(0.0139)	(0.0374)
ERzscore_jt	−0.0319+	−0.139+	−0.0668***	−0.149**
	(0.0172)	(0.0766)	(0.0125)	(0.0506)
**3.South-North**				
ERzscore_it	0.124***	−0.121	0.0876***	−0.0892
	(0.0305)	(0.0804)	(0.0253)	(0.0667)
ERzscore_jt	−0.0258	0.0489	−0.0589*	0.0335
	(0.0251)	(0.125)	(0.0277)	(0.0711)
**4.South-South**				
ERzscore_it	−0.224***	−0.500***	−0.219***	−0.161***
	(0.0430)	(0.0941)	(0.0264)	(0.0488)
ERzscore_jt	0.0830***	0.0537	−0.0170	0.0382
	(0.0240)	(0.0524)	(0.0197)	(0.0302)
Gravity variables	Yes	Yes	Yes	Yes
IJ FE	Yes	Yes	Yes	Yes
Year FE	Yes	Yes	Yes	Yes

Standard errors in parentheses. ***, **, *, + denote significance at 0.1, 1, 5, and 10% levels, respectively. Dependent variable: Similar denotes the export value of similar goods in terms of environmental goods; EG denotes the export value of environmental goods. Columns (1) and (2) are the goods in the list of the APEC; columns (3) and (4) are the goods in the list of the OECD.

Trade flows between the South and North are shown in the third pair. Positive coefficients for the environmental policies in origin *i* in columns (1) and (3) indicate that more stringent environmental regulations in origin *i* (South) promote more trade in similar goods rather than in environmental goods to destination *j* (North). This may be due to the fact that countries in the North tend to import goods from those countries with high environmental standards, but not necessarily environmental goods. For South-South trade flows, which are represented in the fourth pair, strict environmental regulations in origin *i* trigger a less export supply for similar goods. Overall, the stringency of environmental regulations does not promote any trade in environmental goods, but this stringency has effects on the trade patterns of similar goods. In other words, the strict environmental policies do not change the trade relationship for the case of environmental goods, but they do have an effect on similar goods.

### 4.3. The Changes in Environmental Regulation

This subsection seeks to examine whether trade patterns are influenced by the introduction of stricter environmental regulations. On the contrary, what happens if the environmental regulations become laxer? To disentangle the answers to these questions, we create two dummies to capture changes in environmental regulations. We define first a stringent dummy (*Stringen*) to reflect an increase in the proxy for environmental regulations, the z-score, increase in both origin and destination compared to the last period. This dummy takes a value of 1 if both origin and destination countries tighten their environmental regulations, and 0 otherwise. On the contrary, a lax dummy (*Lax*) is introduced to capture a loosening of regulations in both origin and destination.

In this regard, we can control the exporter-time, importer-time, and bilateral fixed effects. The regressions are as follows:


(3) $$\begin{array}{l} Exports\_Similar_{ijt}=exp[\sigma_{1}RTA_{ijt}+\sigma_{2}Changes_{ijt}+\gamma_{it}\\ \;+\alpha_{jt}+\delta_{ij}]\times \varepsilon_{ijt} \end{array}$$

(4) $$\begin{array}{l} Exports\_EG_{ijt}=exp[\sigma_{1}^{\prime }RTA_{ijt}+\sigma_{2}^{\prime }Changes_{ijt}+\sigma_{3}^{\prime }EnvirPro_{ijt}\\ \;+\gamma {âӐŹ}_{it}+\alpha_{it}^{\prime }+\delta {âӐŹ}_{ij}]\times \varepsilon_{ijt}^{\prime } \end{array}$$

where *Changes*_*ijt*_ denotes the proxy for environmental regulations getting stricter (*Stringent* in the table) or laxer (*Lax* in the table).

The results for the case where both origin and destination countries implement stricter environmental regulations than the previous year are shown in Table 4. Negative and statistically significant signs can be observed in columns (2) and (4), for the case of trade in environmental goods. These results can be interpreted as indicating that when environmental regulations are stricter than before in both origin and destination countries, it results in less trade in environmental goods. Moreover, Table 5 shows the case of when both origin and destination countries loosen their environmental policies relative to the previous year. To our surprise, the negative and statistically significant results appear in columns (2), (3), and (4). They indicate that trade tends to be decreased when origin and destination countries both shift toward laxer environmental regulations. To conclude, the trade in environmental goods is very sensitive to the changes in environmental regulations. The trade flows tend to decline in response to a change, regardless of whether the environmental policies in both origin and destination countries become stricter or laxer.

**Table 4:** Turn to stringent.

	**(1)**	**(2)**	**(3)**	**(4)**
	**Similar (APEC)**	**EG (APEC)**	**Similar (OECD)**	**EG (OECD)**
RTA_ijt	−0.0260	0.0246	0.00996	−0.0300
	(0.0219)	(0.0224)	(0.0166)	(0.0192)
Stringent_ijt	0.0258	−0.0419+	−0.00717	−0.0354+
	(0.0213)	(0.0241)	(0.0161)	(0.0182)
EnvirPro_ijt		0.000883		0.000527
		(0.000608)		(0.000433)
Constant	13.11***	13.39***	14.10***	13.55***
	(0.0106)	(0.0105)	(0.00975)	(0.0103)
Observations	333908	406624	382097	447217
IJ IT JT FE	YES	YES	YES	YES

Standard errors in parentheses. ***, **, *, + denote significance at 0.1, 1, 5, and 10% levels, respectively. Dependent variable: Similar denotes the export value of similar goods in terms of environmental goods; EG denotes the export value of environmental goods. Columns (1) and (2) are the goods in the list of the APEC; columns (3) and (4) are the goods in the list of the OECD.

**Table 5:** Turn to lax.

	**(1)**	**(2)**	**(3)**	**(4)**
	**Similar (APEC)**	**EG (APEC)**	**Similar (OECD)**	**EG (OECD)**
RTA_ijt	−0.0266	0.0258	0.0106	−0.0294
	(0.0220)	(0.0224)	(0.0165)	(0.0191)
Lax_ijt	0.0134	−0.0709**	−0.0307+	-0.0425*
	(0.0219)	(0.0237)	(0.0164)	(0.0176)
EnvirPro_ijt		0.000883		0.000527
		(0.000610)		(0.000434)
Constant	13.12***	13.40***	14.11***	13.55***
	(0.0104)	(0.0103)	(0.00971)	(0.0101)
Observations	333908	406624	382097	447217
IJ IT JT FE	YES	YES	YES	YES

Standard errors in parentheses. ***, **, *, + denote significance at 0.1, 1, 5, and 10% levels, respectively. Dependent variable: Similar denotes the export value of similar goods in terms of environmental goods; EG denotes the export value of environmental goods. Columns (1) and (2) are the goods in the list of the APEC; columns (3) and (4) are the goods in the list of the OECD.

## 5. Robustness Check

Robustness checks are developed along two lines to provide a battery of tests for our results. The first one is to replace the z-score by using the law indicators directly, and the second one is to apply another methodology.

Instead of employing the z-score as a proxy for environmental regulations as we do for the main results, we apply an interaction term of the score of law and order effectiveness for each country. The results are shown in Table A8 (in the Appendix). The positive sign and high magnitude of the coefficient for the law score indicate that it has a strong impact on exports. Therefore, the negative impact of the interaction term mitigates this effect when law and order become stricter. In other words, it indicates that when the legal system is more effective, the stricter environmental laws impede trade.

As a second robustness check, we apply a seemingly unrelated regression (SUR) estimation of the gravity model. This methodology allows us to efficiently estimate different regressions that have correlated residuals (Zellner, 1962).


(5) $$\begin{array}{l} \;ln(Exports\_Similar_{ijt})=\gamma_{0}+\gamma_{1}zscoreER_{it}+\gamma_{2}zscoreER_{jt}\\ +\gamma_{3}ln(GDP_{it})+\gamma_{4}ln(GDP_{jt})+\gamma_{5}ln(Y_{it})+\gamma_{6}ln(Y_{jt})+\gamma_{7}RTA\\ +\gamma_{8}ln(DIS_{ij})+\gamma_{9}CON_{ij}+\gamma_{10}ComCurrency_{ijt}+\gamma_{11}ComReli_{ijt}\\ +\gamma_{12}Normal\_Tariff_{ijt}+\gamma_{13}Remoteness_{it}+\gamma_{14}Remoteness_{jt}\\ \;+\beta_{i}+\alpha_{j}+\sigma_{t}+\varepsilon_{ijt} \end{array}$$

(6) $$\begin{array}{l} \;ln(Exports\_EG_{ijt})=\delta_{0}+\delta_{1}zscoreER_{it}+\delta_{2}zscoreER_{jt}\\ +\delta_{3}ln(GDP_{it}+\delta_{4}ln(GDP_{jt})+\delta_{5}ln(Y_{it})+\delta_{6}ln(Y_{jt})+\delta_{7}RTA\\ +\delta_{8}ln(DIS_{ij})+\delta_{9}CON_{ij}+\delta_{10}ComCurrency_{ijt}+\delta_{11}ComReli_{ijt}\\ \;+\delta_{12}EG\_Tariff_{ijt}+\delta_{13}EnvirPro_{ijt}+\delta_{14}Remoteness_{it}\\ \;+\delta_{15}Remoteness_{jt}+\beta_{i}^{\prime }+\alpha_{j}^{\prime }+\sigma_{t}^{\prime }+\varepsilon_{ijt}^{\prime } \end{array}$$

The results for the impact of environmental regulations on trade flows using the SUR estimation are reported in Tables A9, A10 (in the Appendix) for both the APEC and OECD lists, respectively. Table A9 shows that strict environmental policies have a negative impact on trade in environmental goods and similar goods, but this is only statistically significant for the case of environmental goods. The negative signs in Table A10 for the coefficients of environmental regulations in origin *i* show that they reduce exports of both environmental and similar goods. Also, in these results, the magnitude of the variables shows that the regulations have a stronger impact on trade in environmental goods than in similar goods.

## 6. Conclusions

This paper analyzes the impact of environmental policies on trade patterns by comparing trade in environmental goods included in both the APEC and OECD lists and trade in similar goods. The data sample covers 112 exporters and 53 importers from 1989 to 2013. By employing the z-score as a comprehensive for proxy environmental regulations, we include three environmental indicators. A key finding is that strict environmental policies limit trade, which is in line with the previous literature (Jug and Mirza, 2005; Nunez-Rocha and Turcu, 2019).

This study complements existing researches into this trade-environment nexus by highlighting the impact of environmental regulations on the trade pattern, of both environmental goods and similar goods. By comparing the trade in two types of goods, we find that environmental regulations do not help countries to trade more and they even impede trade in environmental goods. We further analyze the link between environmental regulations and environmental goods from the perspective of South-North trade and North-North trade, and so forth. This helps us gain a more in-depth understanding of whether environmental regulations can impact trade relations. The results strongly suggest that stringent environmental policies do not influence the trade relationship for the case of environmental goods but they do so for similar goods. Concerning the changes in environmental regulations, our results indicate that trade in environmental goods is very sensitive to shift in environmental regulations. The trade flows tend to decline when the environmental policies in both origin and destination countries become stricter or laxer. Finally, we find that the stringency of environmental policies in the origin country has a considerably stronger impact on its exports.

Based on this evidence, some policy implications can be highlighted. First of all, governments should pay more attention to the efficiency of environmental regulations. Indeed, strict environmental regulations reduce the trade value, but this stringency does tend to encourage more environmentally-friendly consumption and production. Furthermore, the negotiations between countries could focus on reducing tariff and non-tariff barriers to green trade. Last but not least, this paper does not include the shock of the current situation, such as the epidemic of covid-19. This epidemic changes the global situation from lifestyle of citizens' to international relationships of countries. On these grounds, future interesting research avenue could be on the impact of covid-19 on trade in environmental goods. Moreover, the impact of environmental regulations on global value chains can be also highlighted for future study.

## Data Availability Statement

The raw data supporting the conclusions of this article will be made available by the authors, without undue reservation.

## Author Contributions

ZD collects the data, does the empirical analysis, and writes the manuscript. During the review of manuscript, ZD makes more contribution on reviewing and writing the manuscript. YZ designs the model, finishes the empirical analysis, reviews and writing the manuscript. During the review of manuscript, RZ helps to review the model and revisit the empirical analysis and data checking of the manuscript. All authors contributed to the article and approved the submitted version.

## Funding

ZD would like to thank the financial support received from National Social Science Fund of China, Project number: 20CFX054.

## Conflict of Interest

The authors declare that the research was conducted in the absence of any commercial or financial relationships that could be construed as a potential conflict of interest.

## Publisher's Note

All claims expressed in this article are solely those of the authors and do not necessarily represent those of their affiliated organizations, or those of the publisher, the editors and the reviewers. Any product that may be evaluated in this article, or claim that may be made by its manufacturer, is not guaranteed or endorsed by the publisher.

## Footnotes

^1^The “similar goods” mentioned here refer to products that share the same HS4 commodity code with environmental goods.

^2^Further results are reported in Tables A4–A7 (Appendix).
