# Supplementary material for: The Impact of Environmental Regulations on Trade Flows: A Focus on Environmental Goods Listed in APEC and OECD
Source: Front Psychol. 2021 Nov 26;12:773749. doi: 10.3389/fpsyg.2021.773749 (PMC8662949; doi:10.3389/fpsyg.2021.773749)
Supplement: Supplementary file 1 [file Table_1.pdf]

Appendix

Table 1: Correlation Matrix

	$ln(ExportsNormal)$	$ln(ImportsNormal)$	$ln(ExportsEG)$	$ln(ImportsEG)$	$lnGDP_{it}$	$lnGDP_{jt}$	lnY_{it}	lnY_{jt}	RTA_{ijt}	$lnDIS_{ij}$	CON_{ij}	$ComCurrency_{ijt}$	$ComReli_{ijt}$	EG_tariff_{ijt}	$EnvirPro_{ijt}$	$z\_ERcompos\ o$	$z\_ERcompos\ d$	$Remoteness_i$	$Remoteness_j$
$ln(ExportsNormal)$	1																		
$ln(ImportsNormal)$	0.7474	1																	
$ln(ExportsEG)$	0.7106	0.5795	1																
$ln(ImportsEG)$	0.6249	0.7337	0.4937	1															
$lnGDP_{it}$	0.5588	0.371	0.6139	0.2453	1														
$lnGDP_{jt}$	0.2915	0.4915	0.1251	0.5508	-0.1838	1													
lnY_{it}	0.2635	0.1128	0.4397	0.0578	0.475	-0.1274	1												
lnY_{jt}	0.068	0.1063	0.0113	0.2896	-0.0966	0.384	-0.0457	1											
RTA_{ijt}	0.1615	0.1124	0.1553	0.0945	-0.0071	-0.1535	0.08	-0.024	1										
$lnDIS_{ij}$	-0.1507	-0.1039	-0.1199	0.0019	0.0964	0.3185	0.0208	0.2178	-0.4982	1									
CON_{ij}	0.1528	0.1586	0.0895	0.1098	-0.0585	-0.0477	-0.0855	-0.1079	0.227	-0.3922	1								
$ComCurrency_{ijt}$	0.0759	0.0755	0.071	0.0742	-0.002	-0.0138	0.0214	0.0217	0.0923	-0.1191	0.2131	1							
$ComReli_{ijt}$	-0.0275	-0.0335	-0.1004	-0.0673	-0.1014	-0.072	-0.0552	0.0102	0.2037	-0.2566	0.1345	0.0213	1						
EG_tariff_{ijt}	-0.045	-0.0627	0.0048	-0.1777	0.0292	-0.1471	-0.0032	-0.4025	0.0058	-0.0312	0.037	-0.0241	0.1027	1					
$EnvirPro_{ijt}$	0.0347	0.0352	0.029	0.0336	0.017	-0.0114	0.016	0.003	0.0401	-0.012	0.0161	0.0044	-0.0021	-0.0154	1				
$z\_ERcompos\ o$	0.2266	0.1448	0.3368	0.0739	0.4252	-0.0684	0.5737	-0.0238	0.0479	0.0595	-0.0866	-0.0087	-0.0552	-0.0284	0.0103	1			
$z\_ERcompos\ d$	0.0659	0.0741	0.0243	0.1772	-0.0422	0.2913	-0.0039	0.5088	-0.0264	0.1088	-0.0261	-0.0026	0.0453	-0.2511	-0.0032	0.0074	1		
$Remoteness_i$	-0.383	-0.2501	-0.3733	-0.1839	-0.6819	0.116	-0.3432	0.0627	-0.0215	-0.0271	0.0225	-0.0096	0.013	-0.021	-0.0087	-0.2514	0.0057	1	
$Remoteness_j$	-0.223	-0.3801	-0.1292	-0.3646	0.1227	-0.7341	0.0801	-0.2767	0.0701	-0.2236	0.0195	-0.0061	-0.0106	0.0322	-0.0012	0.0389	-0.1821	-0.071	1

Table 2: Exporting countries list

Albania	Algeria	Angola	Argentina
Armenia	Australia	Austria	Azerbaijan
Bahamas	Bahrain	Bangladesh	Belarus
Bolivia Plurinational State of	Brazil	Brunei Darussalam	Bulgaria
Cameroon	Canada	Chile	China
China Hong Kong SAR	Colombia	Congo	Costa Rica
Croatia	Cyprus	Czech Rep.	Côte d'Ivoire
Denmark	Dominican Rep.	Ecuador	Egypt
El Salvador	Estonia	Ethiopia	Finland
Fmr Sudan	Gabon	Gambia	Germany
Ghana	Greece	Guatemala	Guinea-Bissau
Guyana	Haiti	Honduras	Hungary
Iceland	Indonesia	Iran	Iraq
Ireland	Israel	Jamaica	Japan
Jordan	Kazakhstan	Kenya	Kuwait
Latvia	Lebanon	Libya	Lithuania
Malaysia	Malta	Mexico	Mongolia
Morocco	Mozambique	Myanmar	Netherlands
New Zealand	Nicaragua	Niger	Nigeria
Oman	Pakistan	Panama	Paraguay
Peru	Philippines	Poland	Portugal
Qatar	Rep. of Korea	Rep. of Moldova	Russian Federation
Saudi Arabia	Senegal	Singapore	Slovakia
Slovenia	Spain	Sri Lanka	Suriname
Sweden	Thailand	Togo	Trinidad and Tobago
Tunisia	Turkey	Ukraine	United Arab Emirates
United Kingdom	United Rep. of Tanzania	Uruguay	Venezuela
Viet Nam	Yemen	Zambia	Zimbabwe

Table 3: Importing countries list

Algeria	Argentina	Armenia	Australia
Azerbaijan	Bahrain	Bangladesh	Belarus
Brunei Darussalam	Bulgaria	Canada	China
Croatia	Cyprus	Egypt	Iceland
Indonesia	Israel	Japan	Jordan
Kazakhstan	Kenya	Kuwait	Lebanon
Libya	Lithuania	Malaysia	Malta
Mexico	New Zealand	Nigeria	Oman
Pakistan	Panama	Poland	Qatar
Rep. of Korea	Rep. of Moldova	Russian Federation	Saudi Arabia
Singapore	Slovenia	Sri Lanka	Thailand
Trinidad and Tobago	Tunisia	Turkey	
Ukraine	United Arab Emirates	United Rep. of Tanzania	Venezuela
Zambia	Zimbabwe		

Table 4: North-South flows

	(1) Similar(APEC)	(2) EG(APEC)	(3) Similar(OECD)	(4) EG(OECD)
lnGDP_it	0.156	0.391	-0.181	0.227
	(0.220)	(0.393)	(0.174)	(0.173)
lnGDP_jt	-2.006***	-0.986	-2.545***	-1.631***
	(0.223)	(0.681)	(0.265)	(0.287)
lnY_it	0.296	0.324	0.762***	0.253
	(0.260)	(0.550)	(0.231)	(0.211)
lnY_jt	2.692***	1.903**	3.407***	2.424***
	(0.216)	(0.623)	(0.250)	(0.274)
RTA_ijt	-0.0883*	-0.141	-0.0929*	-0.165***
	(0.0449)	(0.0905)	(0.0441)	(0.0424)
Normaltariff_ijt	-0.00528*		-0.0208***	
	(0.00227)		(0.00515)	
Remoteness_it	2.570***	4.348***	2.473***	1.933***
	(0.585)	(1.213)	(0.594)	(0.586)
Remoteness_jt	-0.681***	2.367***	0.525***	1.335***
	(0.174)	(0.384)	(0.131)	(0.168)
ERzscore_it	-0.0455	-0.0371	-0.242***	-0.0701*
	(0.0279)	(0.0630)	(0.0384)	(0.0345)
ERzscore_jt	0.0597**	-0.00232	0.0600**	0.000164
	(0.0190)	(0.0346)	(0.0225)	(0.0168)
EGtariff_ijt		-0.0156		-0.0166***
		(0.0103)		(0.00352)
EnvirPro_ijt		-0.00445**		-0.00145
		(0.00165)		(0.00120)
Constant	37.00***	11.83	52.88***	29.54***
	(5.539)	(14.70)	(5.942)	(5.663)
Observations	26095	8534	27807	8772
IJ FE	YES	YES	YES	YES
year FE	YES	YES	YES	YES

(a) Standard errors in parentheses
(b) ***, **, *, + denote significance at the 0.1, 1, 5 and 10% level, respectively.
(c) Dependent variable: *Similar* denotes the export value of similar goods in terms of environmental goods; *EG* denotes the export value of environmental goods
(d) Column (1) and (2) are the goods in the list of the APEC; column (3) and (4) are the goods in the list of the OECD
(e) Common currency is omitted in this estimation.

Table 5: North-North flows

	(1)<br>Similar(APEC)	(2)<br>EG(APEC)	(3)<br>Similar(OECD)	(4)<br>EG(OECD)
lnGDP_it	0.372*	1.602*	-0.258*	0.810+
	(0.169)	(0.626)	(0.126)	(0.462)
lnGDP_jt	0.872***	0.808***	-0.363**	0.722***
	(0.183)	(0.217)	(0.140)	(0.190)
lnY_it	0.0776	-0.670	0.846***	-0.189
	(0.185)	(0.687)	(0.150)	(0.519)
lnY_jt	-0.545**	-0.545+	0.773***	-0.335
	(0.208)	(0.307)	(0.168)	(0.231)
RTA_ijt	-0.293**	-0.400***	0.0570	-0.260*
	(0.0993)	(0.117)	(0.100)	(0.109)
ComCurrency_ijt	0.0857**	0	0.143***	0
	(0.0291)	(.)	(0.0234)	(.)
Normaltariff_ijt	-0.0284+		0.0527***	
	(0.0158)		(0.0124)	
Remoteness_it	-1.006*	-0.173	-0.563	1.979*
	(0.493)	(1.238)	(0.436)	(0.808)
Remoteness_jt	-0.0859	2.208***	-0.622+	2.164***
	(0.314)	(0.531)	(0.362)	(0.375)
ERzscore_it	0.0248	-0.157**	-0.119***	-0.0922*
	(0.0223)	(0.0482)	(0.0139)	(0.0374)
ERzscore_jt	-0.0319+	-0.139+	-0.0668***	-0.149**
	(0.0172)	(0.0766)	(0.0125)	(0.0506)
EGtariff_ijt		-0.0348		-0.0343+
		(0.0250)		(0.0197)
EnvirPro_ijt		-0.00421+		-0.00401
		(0.00243)		(0.00248)
Constant	-16.49***	-41.34***	14.16***	-23.87**
	(4.112)	(11.89)	(3.114)	(8.504)
Observations	16291	6290	16807	6462
IJ FE	YES	YES	YES	YES
year FE	YES	YES	YES	YES

(a) Standard errors in parentheses
(b) ***, **, *, + denote significance at the 0.1, 1, 5 and 10% level, respectively.
(c) Dependent variable: *Similar* denotes the export value of similar goods in terms of environmental goods; *EG* denotes the export value of environmental goods
(d) Column (1) and (2) are the goods in the list of the APEC; column (3) and (4) are the goods in the list of the OECD.

Table 6: South-North flows

	(1) Similar(APEC)	(2) EG(APEC)	(3) Similar(OECD)	(4) EG(OECD)
lnGDP_it	-1.339***	-0.744	-1.390***	-1.069
	(0.373)	(0.916)	(0.311)	(0.683)
lnGDP_jt	0.00743	-0.0408	0.0985	0.0357
	(0.172)	(0.339)	(0.144)	(0.184)
lnY_it	2.002***	1.690*	1.965***	1.841**
	(0.345)	(0.855)	(0.286)	(0.657)
lnY_jt	0.392*	0.496	0.121	0.341+
	(0.184)	(0.390)	(0.163)	(0.207)
RTA_ijt	0.0732*	0.0990	-0.0439	0.0227
	(0.0322)	(0.0606)	(0.0379)	(0.0396)
Normaltariff_ijt	-0.0543*		0.00631	
	(0.0217)		(0.0182)	
Remoteness_it	1.306***	1.474*	0.194	-0.144
	(0.258)	(0.598)	(0.128)	(0.470)
Remoteness_jt	0.898+	3.944***	1.432***	2.861***
	(0.475)	(1.064)	(0.412)	(0.625)
ERzscore_it	0.124***	-0.121	0.0876***	-0.0892
	(0.0305)	(0.0804)	(0.0253)	(0.0667)
ERzscore_jt	-0.0258	0.0489	-0.0589*	0.0335
	(0.0251)	(0.125)	(0.0277)	(0.0711)
EGtariff_ijt		-0.0684		-0.0483+
		(0.0439)		(0.0254)
EnvirPro_ijt		0.00128		0.00855**
		(0.00453)		(0.00320)
Constant	30.19***	16.17	32.38***	23.30+
	(8.022)	(19.65)	(6.518)	(13.75)
Observations	26066	10836	28268	11557
IJ FE	YES	YES	YES	YES
year FE	YES	YES	YES	YES

(a) Standard errors in parentheses
(b) ***, **, *, + denote significance at the 0.1, 1, 5 and 10% level, respectively.
(c) Dependent variable: *Similar* denotes the export value of similar goods in terms of environmental goods; *EG* denotes the export value of environmental goods
(d) Column (1) and (2) are the goods in the list of the APEC; column (3) and (4) are the goods in the list of the OECD
(e) The variable of Common currency is omitted in this estimation.

Table 7: South-South flows

	(1)<br>Similar(APEC)	(2)<br>EG(APEC)	(3)<br>Similar(OECD)	(4)<br>EG(OECD)
lnGDP_it	3.104***	3.295**	0.510	3.866***
	(0.549)	(1.229)	(0.466)	(0.700)
lnGDP_jt	0.601	-5.289***	1.113**	-2.184**
	(0.385)	(1.387)	(0.339)	(0.741)
lnY_it	-2.236***	-2.569*	0.330	-3.117***
	(0.538)	(1.187)	(0.449)	(0.679)
lnY_jt	-0.0401	5.150***	-0.478	2.480***
	(0.379)	(1.319)	(0.338)	(0.698)
RTA_ijt	0.120*	0.543***	0.105*	0.0785
	(0.0584)	(0.161)	(0.0504)	(0.0672)
Normaltariff_ijt	-0.0217***		-0.0177***	
	(0.00536)		(0.00529)	
Remoteness_it	2.010***	4.924***	1.023***	3.069***
	(0.328)	(0.636)	(0.255)	(0.415)
Remoteness_jt	-0.825***	-0.110	0.202+	0.622**
	(0.189)	(0.546)	(0.120)	(0.227)
ERzscore_it	-0.224***	-0.500***	-0.219***	-0.161***
	(0.0430)	(0.0941)	(0.0264)	(0.0488)
ERzscore_jt	0.0830***	0.0537	-0.0170	0.0382
	(0.0240)	(0.0524)	(0.0197)	(0.0302)
EGtariff_ijt		0.00861		0.0101+
		(0.0123)		(0.00607)
EnvirPro_ijt		-0.0634**		0.00338
		(0.0204)		(0.00580)
Constant	-70.80***	39.62	-29.93**	-30.99
	(12.16)	(36.25)	(10.88)	(19.57)
Observations	32862	12397	39557	13944
IJ FE	YES	YES	YES	YES
year FE	YES	YES	YES	YES

(a) Standard errors in parentheses
(b) ***, **, *, + denote significance at the 0.1, 1, 5 and 10% level, respectively.
(c) Dependent variable: *Similar* denotes the export value of similar goods in terms of environmental goods; *EG* denotes the export value of environmental goods
(d) Column (1) and (2) are the goods in the list of the APEC; column (3) and (4) are the goods in the list of the OECD.

Table 8: Interaction terms

	(1) Similar(APEC)	(2) EG(APEC)	(3) Similar(OECD)	(4) EG(OECD)
lnGDP_it	0.563***	0.573	0.562***	0.156
	(0.149)	(0.501)	(0.149)	(0.315)
lnGDP_jt	-0.112	-0.270	-0.123	-0.0348
	(0.149)	(0.302)	(0.150)	(0.239)
lnY_it	0.0258	0.522	0.0279	0.715*
	(0.154)	(0.526)	(0.154)	(0.334)
lnY_jt	0.765***	1.001**	0.780***	0.609*
	(0.153)	(0.314)	(0.154)	(0.255)
RTA_ijt	0.410***	0.0811	0.407***	0.324***
	(0.0261)	(0.0594)	(0.0260)	(0.0509)
lnDIS_ij	-0.685***	-0.837***	-0.687***	-0.682***
	(0.0137)	(0.0372)	(0.0137)	(0.0276)
CON_ij	0.524***	0.555***	0.522***	0.651***
	(0.0274)	(0.0870)	(0.0274)	(0.0734)
ComCurrency_ijt	-0.0945*	-0.785***	-0.0977**	-0.303**
	(0.0370)	(0.122)	(0.0369)	(0.104)
ComReli_ijt	0.123***	-0.159	0.117**	0.219+
	(0.0371)	(0.205)	(0.0373)	(0.114)
Normaltariff_ijt	-0.00760**		-0.00822**	
	(0.00277)		(0.00280)	
EnvirPro_ijt	0.0000643	0.00215	0.000112	0.00710*
	(0.000881)	(0.00443)	(0.000878)	(0.00327)
Remoteness_it	0.112**	2.515***	0.119**	0.679*
	(0.0433)	(0.540)	(0.0426)	(0.312)
Remoteness_jt	0.0533	0.872***	0.0583	0.587**
	(0.0615)	(0.234)	(0.0615)	(0.192)
lawnum_o	0.00146**	0.00610**	0.00147**	0.00430**
	(0.000492)	(0.00220)	(0.000494)	(0.00159)
lawnum_d	0.00136**	0.00402*	0.00137**	0.00322**
	(0.000454)	(0.00175)	(0.000457)	(0.00117)
Law and Order	0.0246	0.265***	0.0288	0.0524
	(0.0201)	(0.0693)	(0.0202)	(0.0464)
Law and Order	0.0185	0.100*	0.0202	0.0355
	(0.0213)	(0.0507)	(0.0213)	(0.0462)
lawnum_o*LawandOrder_o	-0.000311**	-0.00135**	-0.000315**	-0.000939**
	(0.000109)	(0.000416)	(0.000109)	(0.000301)
lawnum_d*LawandOrder_d	-0.000307***	-0.000879*	-0.000313***	-0.000669*
	(0.0000929)	(0.000377)	(0.0000932)	(0.000279)
EGtariff_ijt		-0.0237*		-0.0128
		(0.0117)		(0.00854)
Constant	0.721	-4.205	0.914	2.320
	(3.300)	(10.46)	(3.309)	(7.000)
Observations	170218	54624	170218	54624
IJ FE	YES	YES	YES	YES
year FE	YES	YES	YES	YES

(a) Standard errors in parentheses
(b) ***, **, *, + denote significance at the 0.1, 1, 5 and 10% level, respectively.
(c) Dependent variable: *Similar* denotes the export value in similar goods in terms of environmental goods; *EG* denotes the export value in environmental goods
(d) Column (1) and (2) are the goods in the list of the APEC; column (3) and (4) are the goods in the list of the OECD.

Table 9: SUR methodology for environmental goods in APEC list

	(1)	
	ln(Exports_Similar_ijt)	ln(Exports_EG_ijt)
lnGDP_it	0.807***	0.518***
	(0.157)	(0.149)
lnGDP_jt	0.544**	0.425*
	(0.175)	(0.166)
lnY_it	-0.346*	-0.0200
	(0.156)	(0.148)
lnY_jt	0.320	0.171
	(0.181)	(0.171)
RTA_ijt	0.537***	0.552***
	(0.0373)	(0.0353)
lnDIS_ij	-1.353***	-1.294***
	(0.0211)	(0.0200)
CON_ij	0.924***	0.830***
	(0.0664)	(0.0629)
ComCurrency_ijt	-1.227***	-1.548***
	(0.286)	(0.271)
ComReli_ijt	0.758***	0.616***
	(0.0615)	(0.0583)
Normaltariff_ijt	-0.00246	
	(0.00464)	
Remoteness_it	0.847***	0.475***
	(0.149)	(0.141)
Remoteness_jt	-0.173	-0.0115
	(0.133)	(0.126)
ERzscore_it	-0.0335	-0.109***
	(0.0285)	(0.0270)
ERzscore_jt	0.0112	0.0407
	(0.0295)	(0.0278)
EGtariff_ijt		0.00524
		(0.00479)
EnvirPro_ijt		0.000654
		(0.00412)
Constant	-21.05***	-14.26***
	(3.965)	(3.751)
Observations	23559	
r2	0.723	0.756
chi2	61590.4	73124.3
Exporter Dummies	yes	yes
Importer Dummies	yes	yes
Year Dummies	yes	yes

Notes:
(a) Standard errors in parentheses
(b) ***, **, *, + denote significance at the 0.1, 1, 5 and 10% level, respectively.
(c) Dependent variable: $ExportsNormal$ denotes the export value of standard goods; $ExportsEG$ denotes the export value of environmental goods.

Table 10: SUR methodology for environmental goods in OECD list

	(1) ln(Exports_Similar_ijt)	ln(Exports_EG_ijt)
lnGDP_it	0.745***	0.356**
	(0.131)	(0.132)
lnGDP_jt	-0.0531	-0.0297
	(0.156)	(0.157)
lnY_it	-0.243	0.110
	(0.131)	(0.132)
lnY_jt	0.737***	0.639***
	(0.162)	(0.162)
RTA_ijt	0.676***	0.592***
	(0.0335)	(0.0337)
lnDIS_ij	-1.396***	-1.482***
	(0.0187)	(0.0189)
CON_ij	0.861***	0.805***
	(0.0609)	(0.0612)
ComCurrency_ijt	-1.728***	-2.195***
	(0.273)	(0.275)
ComReli_ijt	0.776***	0.640***
	(0.0525)	(0.0528)
Normaltariff_ijt	0.00317	
	(0.00407)	
ERzscore_it	-0.0780**	-0.125***
	(0.0249)	(0.0250)
ERzscore_jt	0.00107	-0.00987
	(0.0262)	(0.0262)
Remoteness_it	0.615***	0.393***
	(0.105)	(0.106)
Remoteness_jt	0.232*	0.344**
	(0.117)	(0.118)
EGtariff_ijt		0.00442
		(0.00442)
EnvirPro_ijt		-0.00148
		(0.00409)
Constant	-8.969**	-1.968
	(3.474)	(3.487)
Observations	27452	
r2	0.754	0.748
chi2	84195.2	81574.2
Exporter Dummies	yes	yes
Importer Dummies	yes	yes
Year Dummies	yes	yes

Notes:
(a) Standard errors in parentheses
(b) ***, **, and * denote significance at 1, 5 and 10% level, respectively
(c) Dependent variable: $ExportsNormal$ denotes the export value of standard goods; $ExportsEG$ denotes the export value of environmental goods.
